# Lipoteichoic Acid from *Lacticaseibacillus rhamnosus* GG Modulates Dendritic Cells and T Cells in the Gut

**DOI:** 10.3390/nu14030723

**Published:** 2022-02-08

**Authors:** Adrián D. Friedrich, Juliana Leoni, Mariela L. Paz, Daniel H. González Maglio

**Affiliations:** 1 Cátedra de Inmunología, Facultad de Farmacia y Bioquímica, Universidad de Buenos Aires, Buenos Aires 1053, Argentina; afriedrich.bioq@gmail.com (A.D.F.); mlpaz@ffyb.uba.ar (M.L.P.); 2 Instituto de Estudios de la Inmunidad Humoral (IDEHU), CONICET—Universidad de Buenos Aires, Buenos Aires 1053, Argentina; jleoni@ffyb.uba.ar

**Keywords:** dendritic cells (DCs), probiotics, TLR-ligands, T cells

## Abstract

Lipoteichoic acid (LTA) from Gram-positive bacteria exerts different immune effects depending on the bacterial source from which it is isolated. *Lacticaseibacillus rhamnosus* GG LTA (LGG-LTA) oral administration reduces UVB-induced immunosuppression and skin tumor development in mice. In the present work, we evaluate the immunomodulatory effect exerted by LGG-LTA in dendritic cells (DC) and T cells, both in vitro and in the gut-associated lymphoid tissue (GALT). During cell culture, LTA-stimulated BMDC increased CD86 and MHC-II expression and secreted low levels of pro and anti-inflammatory cytokines. Moreover, LTA-treated BMDC increased T cell priming capacity, promoting the secretion of IL-17A. On the other hand, in orally LTA-treated mice, a decrease in mature DC (lamina propria and Peyer’s patches) was observed. Concomitantly, an increase in IL-12p35 and IFN-γ transcription was presented (lamina propria and Peyer’s Patches). Finally, an increase in the number of CD103+ DC was observed in Peyer’s patches. Together, our data demonstrate that LGG-LTA activates DC and T cells. Moreover, we show that a Th1-biased immune response is triggered in vivo after oral LTA administration. These effects justify the oral LTA activity previously observed.

## 1. Introduction

One of the major groups of bacteria with demonstrated probiotic activity is the lactic acid bacteria group, particularly the (formerly) *Lactobacillus* species. They are often consumed as foods or dietary supplements and exert their beneficial effects by three main mechanisms: (i) impairing pathogenic bacteria from colonizing the gastrointestinal tract [1], (ii) reinforcing the barrier functions of intestinal epithelium [2,3], and (iii) modulating both the local and systemic immune response by microbe-associated molecular pattern (MAMP) recognition at the gut-associated lymphoid tissue (GALT) [4].

The cell wall of *Lacticaseibacillus*, as with all Gram-positive bacteria, is composed mainly of lipoproteins, peptidoglycan, and lipoteichoic acid (LTA). The latter is considered analogous to lipopolysaccharide (LPS) in Gram-negative bacteria due to its similar biochemical and physiological properties. *Lacticaseibacillus* LTA is an amphiphilic molecule composed of a chain of poly-glycerophosphate with D-Ala substitutions and a hydrophobic anchor that allows its incorporation in the cell membrane [5]. It is an important ligand for the innate immune response [6]. Moreover, LTA is highly immunogenic, and its structure is considerably diverse between bacteria, even at the species level [5]. Thus, depending on the bacterial source from which it has been isolated, LTA can promote diverse immunomodulatory effects. For instance, LTA isolated from *Staphylococcus aureus* (SaLTA) or *Bacillus subtilis,* but not from *Lactiplantibacillus plantarum* (LpLTA), stimulates the secretion of TNF-α in murine macrophages in vitro [7]. Moreover, Kim et al. have demonstrated that LpLTA inhibits SaLTA-induced TNF-α production in THP-1 cells. Moreover, the intraperitoneal administration of LpLTA impaired LPS-induced endotoxic shock in a mouse model [8]. Furthermore, LTA from *Enterococcus faecalis* CECT7121 induces the secretion of IL-6, TNF-α, and IL-12, but not IL-10 in bone marrow-derived dendritic cells (BMDC) [9].

Regardless of its source, LTA interacts with the heterodimer TLR2/6, the co-receptors CD14 and CD36, which ultimately leads to the activation of NF-κB and AP-1 transcription factors through MAPK signaling [10]. Notably, different LTAs isolated from different (formerly) *Lactobacillus* species have shown to promote MAPK signaling with variable intensities. This may be, in part, the cause of the diverse immunomodulatory activity exerted by LTA [11]. However, LTA has not been thoroughly studied at the cellular level; even less have been the efforts to understand the relevance of LTA in vivo.

In previous reports, our group has shown that the oral administration of LTA isolated from the probiotic bacteria *Lacticaseibacillus rhamnosus* GG (LGG) exerts both prophylactic [12] and therapeutic activity [13] in an Ultraviolet B (UVB)-induced skin cancer mouse model. Furthermore, oral LTA has shown to impair UVB-induced immunosuppression by modulating both the innate and the adaptive skin immune system. After UVB exposure, skin challenges (i.e., oxazolone) recruit less inflammatory monocytes and fail to activate T cells. Oral LTA treatment reverts those effects, allowing correct recruitment of monocytes to the inflamed skin and an adequate T cell activation and effector function (for both CD4 and CD8 T cells). The present study aimed to elucidate some of the mechanisms involved in the immunomodulatory effects that oral LTA exerts in the GALT, which likely represents the first immune environment that LTA modifies. To this end, we analyzed in vitro the effects of LTA on the activation, proinflammatory function, and T cell stimulatory capacity of BMDC. We also evaluated the in vivo effects of oral-administered LTA in the total population of DC residents in the lamina propria and the subset of CD103^+^ DC in the Peyer Patches.

## 2. Materials and Methods

### 2.1. Animal Models

Male C57BL/6 (wild type), male B6.Cg-Tg(TcraTcrb)425Cbn/J (OT-II transgenic mice) and female Balb/C mice (The Jackson Laboratories, Bar Harbor, ME, USA) were used at 8–12 weeks of age. They were housed in quarters with a 12 h light–12 h dark cycle and maintained with water and food *ad libitum*. Female mice of the Balb/C strain of 8–10 weeks of age were treated with a single dose or eight doses of 100 µg of LTA (in 100 µL of PBS) by gastric gavage every other day as previously employed by our group [12,13]. A control group administered with 100 µL of PBS was included in each experiment. All procedures were approved by the University of Pittsburgh IACUC and the guidelines established by the Consejo Nacional de Investigaciones Científicas y Técnicas (Argentina) and were approved by the Review Board of Ethics of the Instituto de Estudios de la Inmunidad Humoral.

### 2.2. Lipoteichoic Acid Purification and Administration

LTA was isolated as previously described [14]. LTA preparation was tested for purity by Western blot, as described previously by Weill et al. [12]. LTA (or PBS) oral administration was performed using a feeding needle.

### 2.3. Bone Marrow-Derived Dendritic Cell Differentiation and Stimulation

BMDC were obtained from C57BL/6 (wild type) as previously described [15]. On day six, the BMDC were purified by positive immunomagnetic selection with anti-CD11c Microbeads and MACS Column (Mac, Miltenyi Biotec, Bergisch Gladbach, Germany).

BMDC were seeded in 24-well plates and stimulated with 1, 10, and 50 μg/mL of purified LTA (duplicates). Stimulation was carried out for 24 h at 37 °C in a humidified atmosphere with 5% pCO_2_. Control treatment with PBS as baseline stimulation control and commercial LPS from *Escherichia coli* 011B4 (Sigma-Aldrich, Burlington, MA, USA) in concentration 0.2 µg/mL as positive control were included. The cells were recovered for analysis by flow cytometry, and the cell-free supernatants were preserved with a protease inhibitor cocktail (Sigma, Burlington, MA, USA) and frozen at −20 °C for subsequent cytokine quantification.

### 2.4. Expression of MHC Class-II and CD86 and Cytokine Secretion by LTA-Treated BMDC

BMDC were cultured in the presence or not (control) of LTA (1, 10, and 50 µg/mL), *E. coli* LPS (0.2 µg/mL), for 24 h. Subsequently, cells and culture supernatants were collected to analyze DC maturation phenotype and cytokine secretion, respectively. For FACS analysis of DC phenotype, cells were incubated with AFluor700 CD86 (GL1), BV421 CD11c (N418) (BD Bioscience, Franklin Lakes, NJ, USA), PE-Cy7 IA (AF6-120.1, BioLegend, San Diego, CA, USA) according to manufacturer protocols. Following washing, cells were fixed with 2% paraformaldehyde and stored at 4 °C in the dark until FACS analysis.

Quantification of IL-12p70, IL-1β, and IL-10 (EPX01A-26004-901, BMS6002TEN and 88-7105-88, eBioscience, Thermo Fisher Scientific, Waltham, MA, USA), IL-6, and TNF-α (555240 and 555268 BD Bioscience, Franklin Lakes, NJ, USA) was performed following the instructions of each manufacturer in BMDC culture supernatant.

### 2.5. Antigen-Specific T Cell Proliferation

CD4^+^ OT-II cells, which express a recombinant TCR specific for an OVA peptide, were isolated from spleens of 8 to 10 weeks old mice and purified by negative selection using an Untouched Mouse CD4 Cell Kit (Dynabeads, Thermo Fisher, Waltham, MA, USA) according to the manufacturer’s protocols and labeled with carboxyfluorescein-succinimidyl ester (CFSE, 1 μM) (Thermo Fisher, Waltham, MA, USA). CD4^+^ OT II cells (responders) were then co-cultured (1:1 ratio) with BMDC (stimulators) pretreated or not (negative control) with LTA (50 µg/mL) or LPS (0.2 µg/mL) (positive control), in triplicates using 96 round-bottom well plates (Corning Costar, Sigma-Aldrich, Burlington, MA, USA) for 4 days in the presence or not of a mixture of OVA (1 mg/mL) (Sigma-Aldrich, Burlington, MA, USA) and OVA_323-339_ peptide (0.1 mg/mL) as antigen source to induce specific T cell activation.

Following culture, cells were separated and immunostained with APC-CD4 Ab (RM4-5, BD Bioscience, Franklin Lakes, NJ, USA), labeled with the eFluor780-Fixable Viability Dye (FVD) (eBioscience, Thermo Fisher, Waltham, MA, USA), and analyzed by FACS.

Detection of the T cell cytokines (IFN-γ, IL-17A, IL-13, and IL-5) in co-cultures supernatants was performed by ELISA (555138 and 555236 -IFN-γ and IL-5- BD Bioscience, USA; M17AF0 and DY413 -IL17A and IL-23- R&D Systems, Minneapolis, MN, USA), according to manufacturer’s protocols.

### 2.6. Analysis of Cytokine Transcripts in the GALT

Gene transcription analysis was performed with four groups of 5 BALB/c mice each, three groups received a single dose of 0.1 mg LTA by gavage, and the remaining was treated with PBS as control. The animals treated with LTA were euthanized 16, 24, and 48 h after oral administration, whereas the group treated with PBS was euthanized at 24 h. Subsequently, the entire small intestine (SI) free of Peyer’s patches (PP), PP, and mesenteric lymph nodes (MLN) were removed. The organs were homogenized using a tissue tearor (Thomas Scientific, Swedesboro, NJ, USA) in 1 mL of Trizol (Thermo Fisher Scientific, Waltham, MA, USA). The samples were frozen at −80 °C in Trizol for preservation. The total RNA was extracted from each sample. Next, reverse transcription was performed to obtain the corresponding cDNA using the M-MLV enzyme (Promega Corporation, Madison, WI, USA).

Reverse transcription PCR (RT-PCR) was performed using the enzyme Taq polymerase (InBioHighWay, Tandil, Buenos Aires, Argentina) for semi-quantitative evaluation of transcripts of the following genes: IL-12p35, IFN-γ, IL-10, TNF-α, IL -6, TGF-β, and IL-1β. In addition, the constitutive expression of GAPDH was used as an amplification control. The sequences of the primers used are described in Table 1.

The PCR products were resolved by electrophoresis on a 2.5% agarose gel stained with SYBR Safe probe (Invitrogen), visualized using a blue-light LED transilluminator (Maestrogen, Las Vegas, NV, USA), and photographed with an Olympus digital camera (QColor 3). The images obtained were analyzed with IMAGE J software. The results, as a relative expression regarding GAPDH, were expressed as mean ± SD.

### 2.7. Cell Phenotype in the GALT

Two groups of 7 female Balb/C mice each, administered with eight doses of LTA or PBS, were euthanized 24 h after the last dose. The SI, PP, and MLN were removed. To prepare Lamina propria single-cell suspensions, the SI was cut lengthwise and washed in PBS. Then, SI was cut into small pieces and incubated in 2 mM EDTA in Hank’s buffer or HBS (Hank’s buffer solution) with 5% FBS for 20 min at 37 °C while stirring. The tissue was then filtered with a nylon strainer, and the procedure was repeated once more. In the end, the filtered tissue was incubated with 1.5 mg/mL type IV collagenase (Worthington, USA) and 40 µg/mL DNase I (Worthington, Columbus, OH, USA) in HBS with 5% FBS for 15 min at 37 °C while stirring. At the end of the incubation, it was stirred with a vortex for 20 s. The suspension obtained was filtered through a 70 µm membrane, centrifuged at 300× *g* for 10 min at 4 °C, and resuspended in 1 mL of 5% FBS in HBS. Finally, cells were labeled with the following directly conjugated Abs PE-CD11c (N418), AFluor647-IA/IE (M5.114.15.2), FITC-CD86 (GL1) and with biotin-CD40 (1C10) and biotin-CD103 (2E7) the two latter followed by a secondary PE-Cy7streptavidin, and with (7-aminoactinomycin D (7AAD) (eBioscience, Thermo Fisher, Waltham, MA, USA).

Single-cell suspensions from the PP were obtained by incubating 15 min with 5 mL of 1.5 mg/mL collagenase type IV and 40 μg/mL DNase I in HBSS with 5% FBS for 15 min at 37 °C with stirring. The samples were then filtered using a 70 μm membrane and pressed with a rubber plunger to disrupt the remaining tissue. Immediately, 5 mL of 2 mM EDTA in HBS with 5% FBS were added for 10 min at 37 °C with stirring. Samples were incubated for 5 min at room temperature and under stirring. Finally, the samples were filtered with 40 μm filters, centrifuged at 300× *g* for 10 min at 4 °C, and the samples were resuspended in 1 mL of 5% SFB in HBS and stored on ice until labeled with the antibodies mentioned above. The cellular suspensions of the MLN were obtained by mechanical disruption, pressing the tissue with a rubber plunger on 70 μm filters first, then being filtered through 40 μm membranes. The suspensions were centrifuged at 300× *g* for 10 min at 4 °C, resuspended in 5% FBS in HBS, and kept cold until labeled with the antibodies mentioned above.

The cell suspensions were acquired in a FACSAria II cytometer (BD Bioscience, Franklin Lakes, NJ, USA). The data were analyzed using FlowJo^®^ v7.6.2 software.

### 2.8. Statistical Analysis

GraphPad Prism 5.0 software (GraphPad Software, San Diego, CA, USA) was used to elaborate graphs; GraphPad InStat 3.06 software (GraphPad Software, San Diego, CA, USA) was used for statistical analysis.

To compare means between 2 groups, Student’s *t*-test with one or two tails was used, provided that the populations had normal distributions and the Mann–Whitney U test with one or two tails was used for non-normal distributions. In experimental designs with more than two experimental groups, an ordinary ANOVA test and a Kruskal–Wallis or Tukey or Dunnett post-test were used to compare groups against a single control group. Variations that showed a significance of less than 0.05 (* *p* < 0.05; ** *p* < 0.01, *** *p* < 0.001 and **** *p* < 0.0001; ns: non-significant differences) were considered significant.

## 3. Results

### 3.1. LTA from LGG Promotes the Activation of BMDC

**Figure 1:**
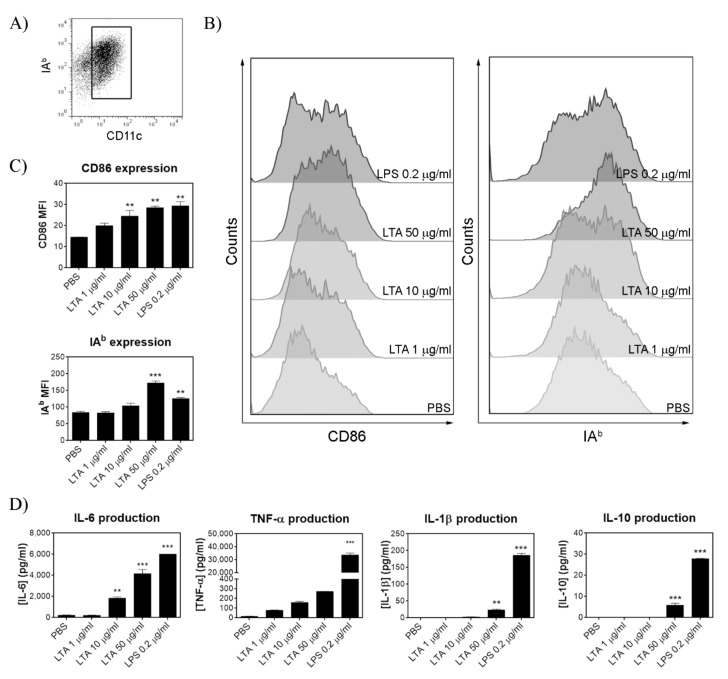
LTA activates BMDC by inducing co-stimulatory molecule expression and cytokines release. (**A**) Gating strategy to identify DC for their expression of MHC class II (PECy7-IAb Ab) and CD11c (FITC-CD11c Ab). (**B**) Representative histograms corresponding to CD86 and MHC class II surface expression levels on BMDC after control (PBS), LTA, and LPS treatment. (**C**) Bar diagrams are mean fluorescence intensity (MFI) ± SD. (**D**) Cytokines (IL-6, TNF-α, IL-1β, and IL-10) production measured by ELISA in cell culture supernatants after 24 h of LTA, LPS, or control treatment. Bar diagrams are means ± SD. (*n* = 2/group). ** *p* < 0.01, *** *p* < 0.001 vs. PBS (ordinary ANOVA and Tukey’s multiple comparisons test).

To assess the immunomodulatory effect of LTA in DC in vitro, we analyzed the activation phenotype and secretion of pro-inflammatory cytokines by BMDC cultured in the presence or not of different concentrations of LTA. The concentration of 50 μg/mL of LTA increased the expression of surface MHC-II (IA^b^) and CD86 in BMDC to a similar extent of that observed in BMDC stimulated with 0.2 µg/mL *E. coli* LPS (positive control) (Figure 1A). Following that, we analyzed the cytokine secretion profile in LTA-treated BMDC by ELISA. At the optimal dose of 50 μg/mL, LTA significantly increased the secretion of the pro-inflammatory cytokines IL-1β, IL-6, and TNF-α and the anti-inflammatory cytokine IL-10, as expected (Figure 1B). However, the ratio between the anti-inflammatory cytokine IL-10 and the pro-inflammatory cytokine TNF-α was higher in LTA (50 µg/mL) than in LPS (47 vs. 1207, respectively), showing a certain degree of bias towards a regulatory or anti-inflammatory activity. Even though LTA isolated from other bacterial sources induced IL-12 secretion by BMDC [9,16,17], LTA from LGG was unable to upregulate neither IL-12p70 nor IL-12p40 secretion in our working conditions (data not shown). Thus, these results demonstrate that LTA is capable of activating BMDC by inducing both MHC-II and CD86 expression as well as cytokine release.

### 3.2. LTA Treatment Increases T Cell Priming Capacity of BMDC

**Figure 2:**
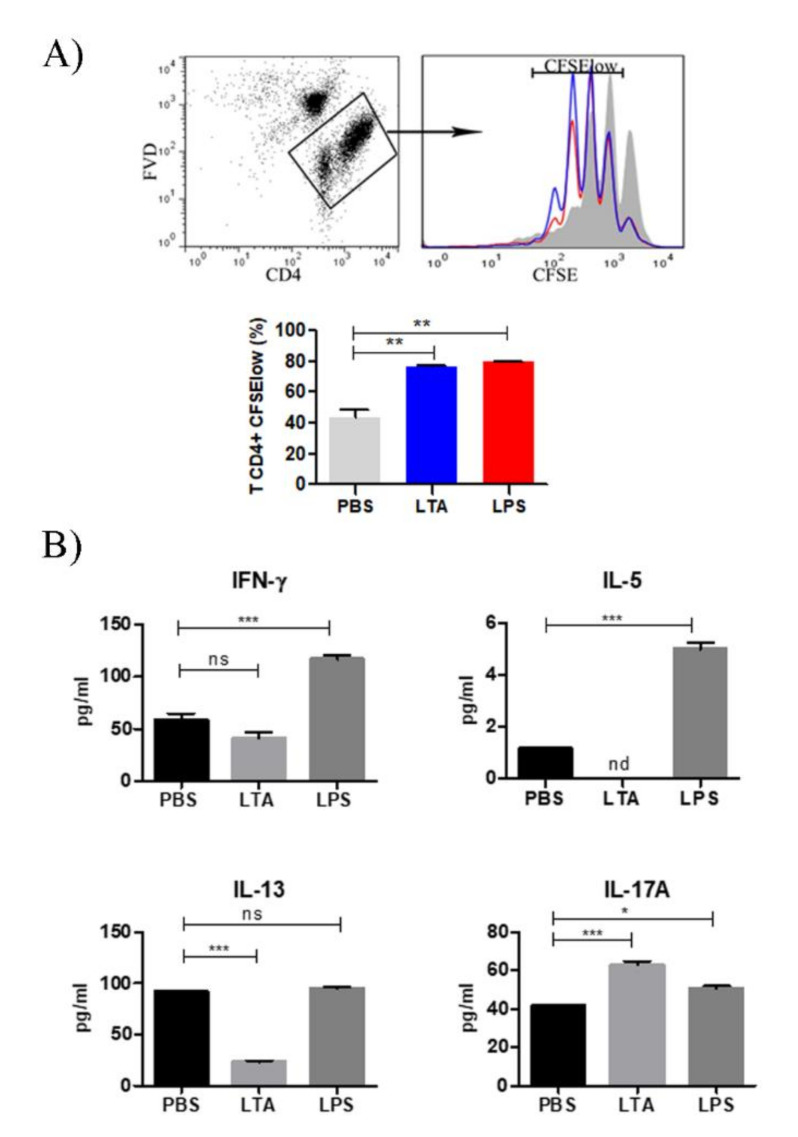
LTA-treated BMDC show an increased priming capacity and induce T cells to secrete IL-17A in vitro. (**A**) Gating strategy to identify FVD- (fixable viability dye) viable CD4^+^ cells (APC-CD4 Ab). Representative histograms of CFSE dilution of viable OT-II CD4+ T cells co-cultured for four days with control (solid), LTA (blue line), and LPS (red line)-treated BMDC. Proliferated cells are indicated as CFSElow. Bar diagrams are means ± SD. (*n* = 3/group). * *p* < 0.05, ** *p* < 0.01, *** *p*< 0.001 (ordinary ANOVA and Kruskal–Wallis post-test). (**B**) Cytokines (IFN-γ, IL-5, IL-13, and IL-17A) production measured by ELISA in co-culture supernatants.

The ability of LTA to modulate the T cell stimulatory function of BMDC was analyzed in co-cultures of LTA-treated BMDC and CD4^+^ OT-II T cells. LTA-treated BMDC induced a higher proliferation of OT II cells than that observed with control BMDC. LTA produced this effect in a similar manner to that observed with lower concentrations of *E. coli* LPS (Figure 2A).

Then, we compared by ELISA the amounts of IFN-γ (Th1), IL5, and IL-13 (Th2), and IL-17A (Th17) secreted by responder T cells stimulated with LTA- or LPS-treated BMDC. LTA and LPS significantly increased IL-17A secretion, but only LPS-treated BMDC augmented IFNγ, IL-5, and IL13 (Figure 2B). These results indicate that LTA from LGG increases the BMDC capacity to prime naïve CD4 T cells to a comparable extent to that induced by LPS-treated BMDC. However, LTA-treated BMDC induce a particular profile of cytokines secreted by the responder T cells, which is different from that induced by LPS-treated BMDC.

### 3.3. Oral LTA Modifies Cytokine Transcription in the GALT

Orally administered LTA is likely sensed by immune cells of the GALT. Therefore, we analyzed the cytokine transcripts in the SI, PP, and MLN, 16, 24, and 48 h after a single dose of LTA. We determined IL-12p35, IFN-γ, IL-10, TNF-α, IL-6, TGF-β, and IL-1β transcripts in the three organs, using GAPDH as the housekeeping gene. The cytokines that showed statistically significant differences are presented.

**Figure 3:**
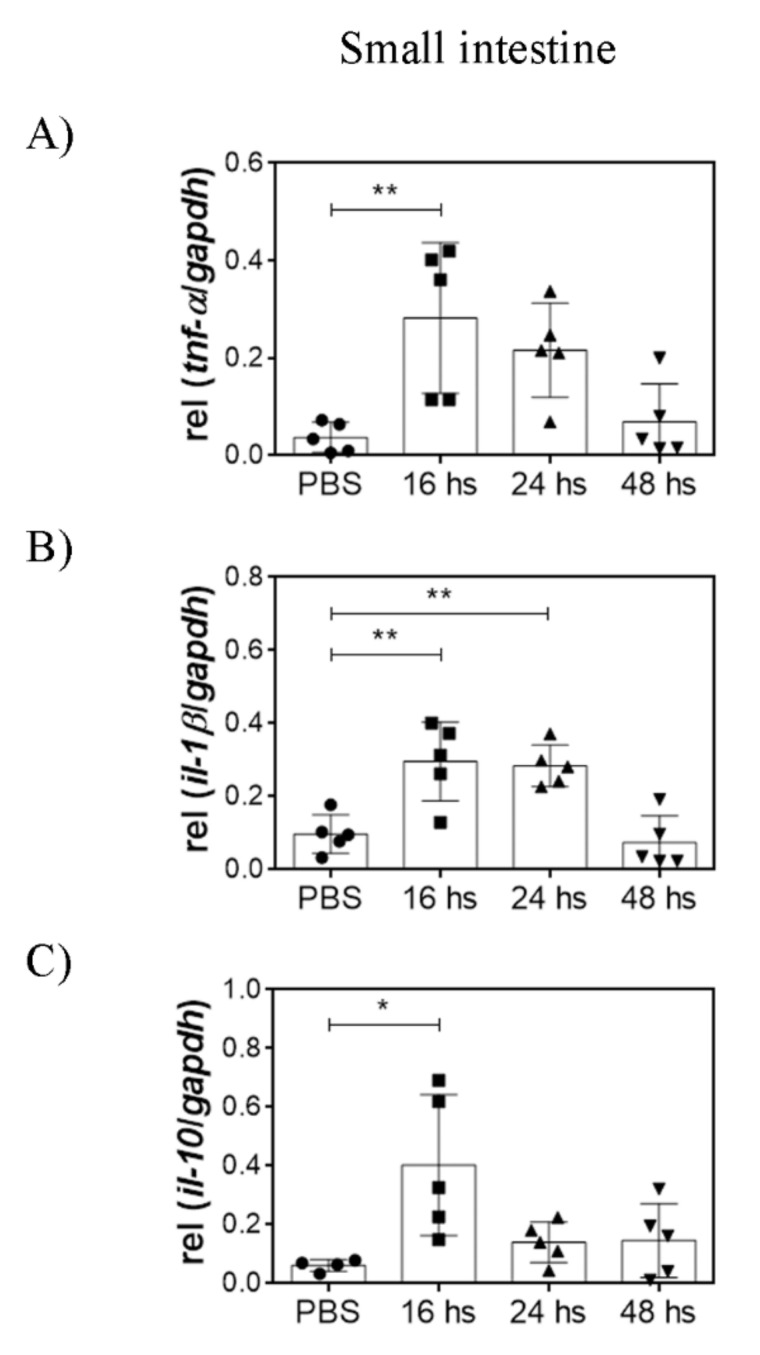
Orally administered LTA modulates cytokine transcription in the GALT. Cytokine mRNA transcription levels were evaluated in small intestine homogenates after mice were orally administered with 100 µg of LTA. Measurements were performed at 16, 24, and 48 h after stimuli. The control group was treated with PBS, and the measurement was performed at 24 h. (**A**) TNF-α transcription, (**B**) IL-1β transcription and (**C**) IL-10 transcription. The results are shown relative to GAPDH and expressed as mean ± SD (*n* = 5/group). * *p* < 0.05, ** *p* < 0.01 (ordinary ANOVA and Dunnett’s post-test comparing each experimental group against control group). ● = PBS-treated mice (control group), ■ = LTA-treated mice sacrificed 16 hs after administration, ▲ = LTA-treated mice sacrificed 24 hs after administration, ▼ = LTA-treated mice sacrificed 48 hs after administration.

**Figure 4:**
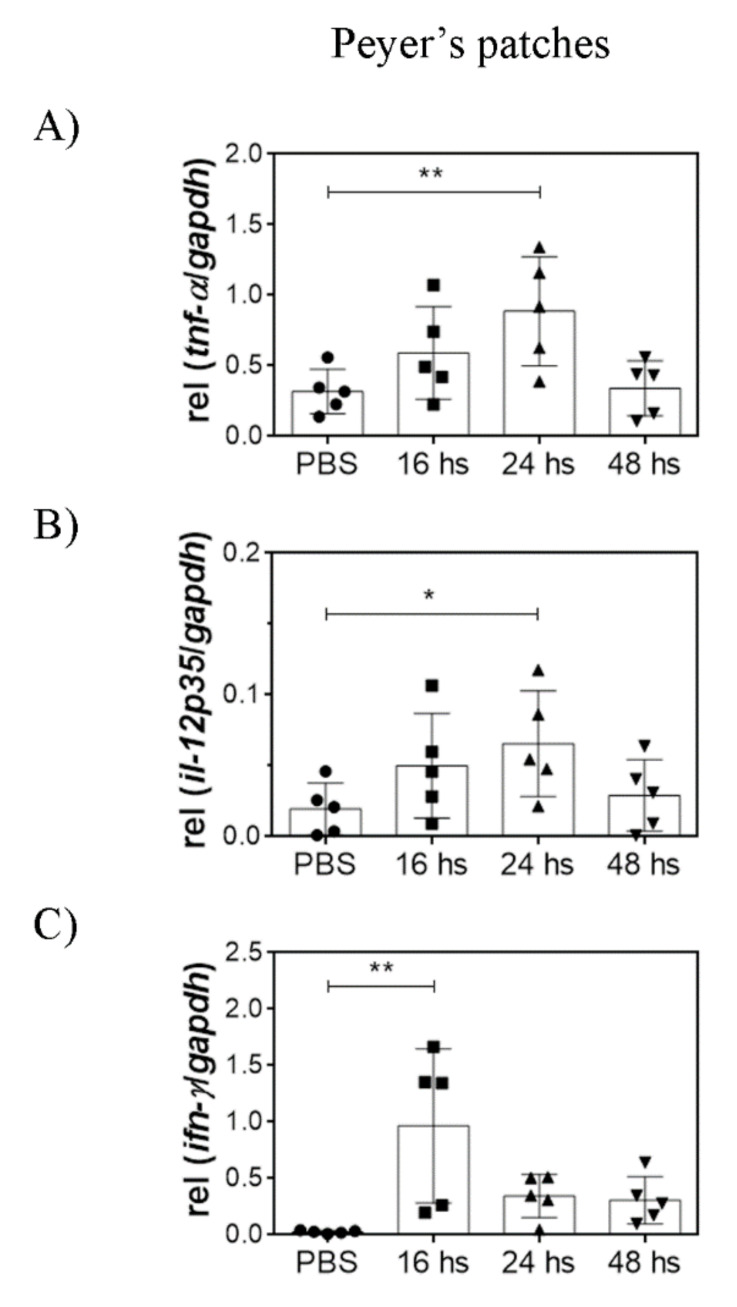
Orally administered LTA modulates cytokine transcription in the GALT. Cytokine mRNA transcription levels were evaluated in Peyer’s patch homogenates after mice were orally administered with 100 µg of LTA. Measurements were performed at 16, 24, and 48 h after stimuli. The control group was treated with PBS, and the measurement was performed at 24 h. (**A**) TNF-α transcription, (**B**) IL-12p35 transcription, and (**C**) IFN-γ transcription. The results are shown relative to GAPDH and expressed as mean ± SD (*n* = 5/group). * *p* < 0.05, ** *p* < 0.01 (ordinary ANOVA and Dunnett’s post-test comparing each experimental group against the control group). ● = PBS-treated mice (control group), ■ = LTA-treated mice sacrificed 16 h after administration, ▲ = LTA-treated mice sacrificed 24 h after administration, ▼ = LTA-treated mice sacrificed 48 h after administration.

**Figure 5:**
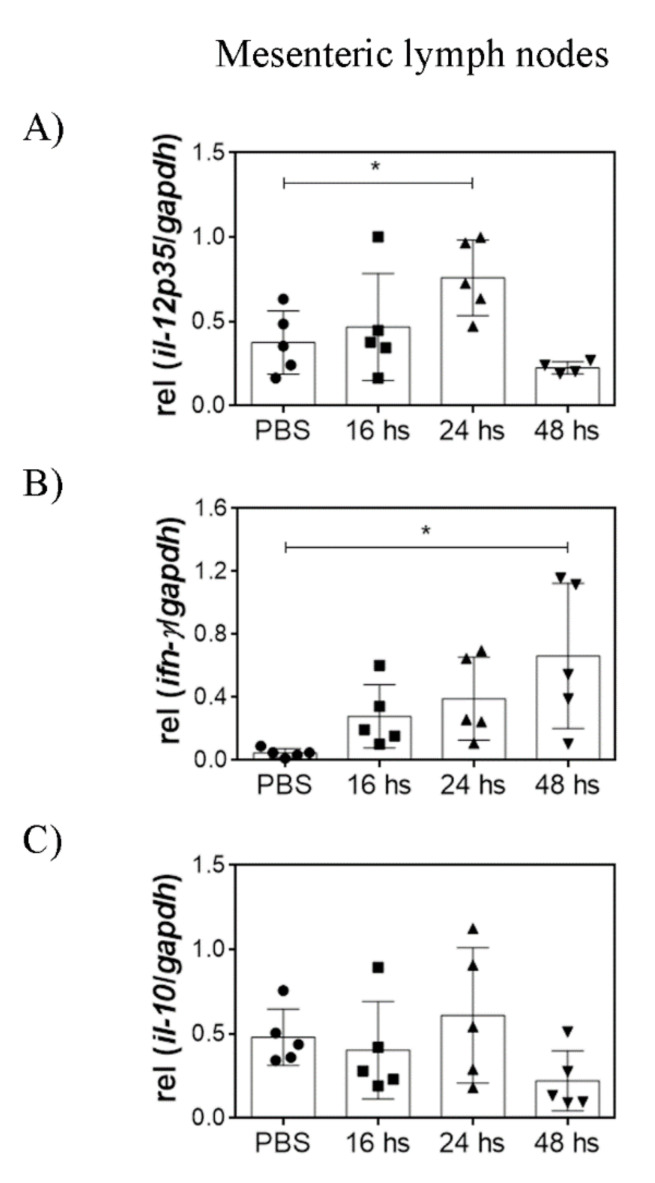
Orally administered LTA modulates cytokine transcription in the GALT. Cytokine mRNA transcription levels were evaluated in mesenteric lymph nodes homogenates after mice were orally administered with 100 µg of LTA. Measurements were performed at 16, 24, and 48 h after stimuli. The control group was treated with PBS, and the measurement was performed at 24 h. (**A**) IL-12p35 transcription, (**B**) IFN-γ transcription, and (**C**) IL-10 transcription. The results are shown relative to GAPDH and expressed as mean ± SD (*n* = 5/group). * *p* < 0.05 (ordinary ANOVA and Dunnett’s post-test comparing each experimental group against control group). ● = PBS-treated mice (control group), ■ = LTA-treated mice sacrificed 16 h after administration, ▲ = LTA-treated mice sacrificed 24 hs after administration, ▼ = LTA-treated mice sacrificed 48 h after administration.

In the SI, TNF-α, IL-1β, and IL-10 transcripts increased 16 h after treatment and decreased after 48 h. Notably, the increase in IL-1β transcripts persisted during 24 h, whereas TNF-α and IL-10 increment was lost more rapidly (Figure 3). No differences were observed in the levels of the other cytokines by LTA treatment (data not shown). High transcription levels of TNF-α were also observed in the PP after 24 h (Figure 4). In contrast to in vitro observations using BMDC, high levels of IL-12p35 were detected both in the PP and in the MLN after 24 h (Figure 5). Moreover, high IFN-γ transcription levels were detected in the PP as soon as 16 h after treatment and in the MLN 48 h after treatment.

These results show that oral administration of purified LTA activates the immune response in the GALT, observed as an inflammatory state.

### 3.4. LTA Promotes the Reduction in Mature DC in the Lamina Propria, but It Increases the Population of CD103^+^ DC in the PP

**Figure 6:**
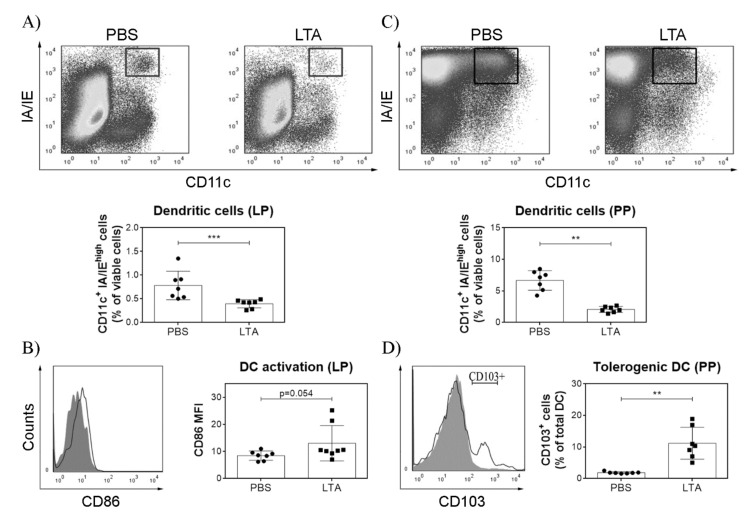
LTA promotes mature DC reduction in the lamina propria whereas it increases CD103^+^ DC presence in the Peyer´s patches. Representative dot-plots of pre-gated viable single CD11c^+^ (PE-CD11c Ab) and MHC class II (Alexa647-IA/IE Ab) with high expression (IA/IEhigh) mature DC in LTA-treated and control (PBS) mice (**A**) lamina propria (LP) and (**C**) Peyers’s patches (PP). Bar diagram shows mean percentages of mature DC ± SD. (**B**) Representative overlaid histograms of CD86 (FITC-CD86 Ab) surface expression on mature DC in the lamina propria of control and LTA-treated mice. Results are expressed as mean fluorescence intensity ± SD. (**D**) Representative overlaid histograms of CD103 (Biotin-Stp-PE-Cy7-CD103 Ab) surface expression on mature DC in the Peyer’s patches of control and LTA-treated mice. Bar diagram shows mean percentage of CD103^+^ DC ± SD (*n* = 7/group). ** *p* < 0.01 *** *p* < 0.001 Student’s *t*-test. ● = PBS-treated mice (control group), ■ = LTA-treated mice sacrificed.

In order to evaluate the effects of LTA on cell populations and phenotype in the GALT, we performed a multiple administration approach. Twenty-four hours after the last oral dose, LTA induced a significant reduction in mature CD11c^+^ MHC-II (IA/IE)^high^ DC in the LP compared with control animals (Figure 6A). Notably, LTA-treated animals showed a trend (*p* = 0.054) to slightly increase CD86 expression on the remaining mature DC population in the LP, suggesting that while being activated, they still have not migrated through lymph vessels (Figure 6B). Surprisingly, although total CD11c^+^ MHC-II (IA/IE)^high^ cells in the PP were also diminished after LTA treatment (Figure 6C), CD103^+^ DC increased in this tissue, suggesting that LTA may promote a homeostatic local response in the GALT (Figure 6D). We could not observe differences in CD11c^+^ MHC-II (IA/IE)^high^ DC populations in the MLN (Figure S1).

## 4. Discussion

Several studies have focused on the consumption of fermented foods and their benefits to human health [18,19,20]. They include the notion that probiotic microorganisms are primarily responsible for those benefits. However, understanding the mechanisms involved in the complex network of interactions that are established between probiotics, the microbiota, and the host, has recently gained noticeable interest. In this regard, LTA has been proposed as a critical molecule in probiotic-host’s immune system interaction.

The immune system acts through innate and adaptive mechanisms due to molecules recognition, cytokine secretion, surface molecules transcription, and cell–cell interactions, among other mechanisms. We aimed to study the role of oral administration of isolated LTA from *L. rhamnosus* GG on immunity, studying some of those mechanisms, focusing on DC and T cells, and using in vitro and in vivo approaches.

Employing BMDC cultures, we show that LTA induced the maturation of DC by increasing the expression of MHC-II and the co-stimulatory molecule CD86. In comparison with LPS, LTA was notably less stimulating since it produced the maturation of the BMDC at a concentration of 50 µg/mL, whereas LPS induced a similar effect with only 0.2 µg/mL. As T cell activation depends on the expression of co-stimulatory molecules and the type and quantity of cytokines released, we evaluated secreted molecules in the culture. LTA from LGG stimulated the secretion of the pro-inflammatory cytokines IL-6, TNF-α, and IL-1β in a dose-dependent manner, although it did not induce detectable amounts of IL-12p70. On the contrary, the anti-inflammatory cytokine IL-10 was secreted in low quantities only with the highest concentration of LTA. Compared with *E. coli* LPS, LTA from LGG showed a milder BMDC-activation capacity. These results are in line with previous reports, which indicate that LTA isolated from different sources are in all cases less potent than LPS from *E. coli* as inflammatory inducers [7,21]. Interestingly, LTA-treated BMDC secreted a ratio of IL-10/TNF-α much higher than LPS, contributing to the idea of a less potent inflammatory capacity and a possible regulatory role of LTA from LGG. According to this observation, it has been recently published that LTA from *L. plantarum* is crucial modulating TLR-3-triggered inflammatory response, promoting the decrease in inflammatory cytokines after poly(I:C) challenge in vivo [22].

Regarding T cell priming, our in vitro experiments showed that LTA-treated BMDC stimulated the proliferation of CD4^+^ T cells in the presence of the antigen. The effect was similar to that produced by LPS. In terms of cytokines secretion, the absence of IFN-γ in the co-cultures’ supernatant is consistent with the inability of LTA-stimulated BMDC to secrete IL-12p70, confirming that LTA from LGG does not favor a Th1 profile in vitro. Moreover, the diminished and non-detectable levels of IL-13 and IL-5, respectively, suggest that neither a Th2 profile is induced. On the contrary, the increase in IL-17A levels in the co-culture suggests that a Th17 profile may be induced. This hypothesis correlates with the secretion of IL-1β and IL-6 by LTA-treated BMDC since they are fundamental cytokines for the differentiation of T cells into Th17 [23]. It is important to highlight that the BMDC cultures are composed by heterogeneous DC populations, and they also contain macrophages precursors that can also respond to bacterial stimuli [24].

To study the effects of LGG LTA on DC and the GALT, we used an oral administration mice model. In accordance with in vitro results, after a single oral LTA administration, we detected a pro-inflammatory response characterized by TNF-α and IL-1β transcription together with the transcription of the anti-inflammatory cytokine IL-10 in the small intestine. These results at a short time after stimuli administration are consistent with a direct recognition of the LTA in the gut lumen, possibly due to TLR2 expression in epithelial cells and/or intraepithelial T lymphocytes [25]. Moreover, the direct recognition of LTA can also take place in Peyer’s patches. The transcription of pro-inflammatory TNF-α was also observed in this organ, demonstrating that oral LTA directly activates innate immunity. It is worth noticing that the inflammatory potency of LTA is less than those observed for LPS, suggesting that the observed responses do not imply a risk of intense intestinal inflammation.

Concerning adaptive immunity activation, and despite our in vitro observations, oral LTA was able to increase both IL-12p35 and IFN-γ transcription in Peyer’s patches and mesenteric lymph nodes. These results suggest that a Th1 profile may be induced in vivo in the GALT by oral LTA. The differences between in vitro and in vivo results are probably due to interactions with other cell types present in the microenvironment that can also modulate the observed response. Unfortunately, there are no reports of the cytokine expression profile in the GALT of healthy animals after oral administration of TLR2 agonists. We speculate that γδ T cells may play a crucial role in LTA local effects since these cells populate the small intestine and have been implicated in the production of IFN-γ after TLR2 agonists recognition [26]. Moreover, it has been established that DC pre-incubated with activated γδ T cells enhance the production of IFN-γ by alloreactive T cells in a mixed lymphocyte reaction [27] and that LPS-treated γδ T cells are able to induce IL-12p70 secretion by DC in vitro [28]. The presence of γδ T cells could explain, at least partially, the early peak of IFN-γ in Peyer’s parches and the subsequent increase in IL-12 observed in this tissue after LTA treatment.

To analyze the effects on DC present in the gut, we focused on CD11c^+^ MHC-II (IA/IE)^high^ cells as a group of mature DC since these cells are composed by complex and heterogenic cell populations [29]. After multiple administrations, LTA-treated mice showed a decrease in the population of mature DC in lamina propria, probably due to the migration of these cells, as previously shown after oral LPS administration [30]. In agreement with this hypothesis, remaining mature DC showed a strong tendency to express higher levels of CD86, indicating a greater degree of activation. Unexpectedly, the percentage of mature DC in secondary lymphoid organs did not increase; it decreased in the Peyer’s patches and remained unaffected in the mesenteric lymph nodes. Considering our previous findings regarding the effects of oral LTA on the skin [13], it can be speculated that these activated cells are induced to migrate through lymph and blood to distant inflamed tissues. Besides DC activation, it should be noted that CD103^+^ DC effectively increased in the Peyer’s patches. As this DC subpopulation is a critical modulator in the induction of regulatory T cells in the GALT in the steady-state [31,32], our results suggest that LTA may play a role in gut homeostasis. However, CD103 is also a marker for conventional/classical DC type 1 [33], and these cells are known for their ability to prime T cells and activate anti-tumor immunity [34]. The expansion of these cells observed in Peyer’s patches may also be related to the anti-tumor effect observed in mouse skin [12].

As we have previously shown, orally administered LTA from LGG affects adaptive immune responses in distant organs, such as the skin. This effect is related to both a prophylactic and therapeutic anti-tumoral activity in skin cancer models and UV-induced immunosuppression [12,13]. This article demonstrates that LGG LTA activates DC promoting a soft inflammatory response with the consequent T cell activation. Even though the complex interactions that take place in vivo are difficult to reproduce using in vitro models, we show that a Th1 biased immune response is triggered after oral LTA administration. This effect justifies the anti-tumoral activity previously observed. It would be of great interest to further evaluate the mechanisms implicated in the crosstalk between the GALT and the skin immune system that could explain how orally administered probiotic-derived molecules impact distant organs.

## Figures and Tables

**Table 1:** Nucleotide sequences of primers used in the RT-PCR.

Gen	5′-3′ *Forward Primer Sequence*	5′-3′ *Reverse Primer Sequence*
**IL-12p35**	TACTAGAGAGACTTCTTCCACAACAAGAG	TCTGGTACATCTTCAAGTCCTCATAGA
**IFN-γ**	TGCATCTTGGCTTTGCAGCTCTTC	GGGTTGTTGACCTCAAACTTGGCA
**IL-10**	ATGCTGCCTGCTCTTACTGACTG	CCCAAGTAACCCTTAAAGTCCTGC
**TNF-α**	GGCAGGTCTACTTTGGAGTCATTGC	ACATTCGAGGCTCCAGTGAATTCGG
**IL-6**	CGTGGAAATGAGAAAAGAGTTGTGC	ATGCTTAGGCATAACGCACTAGGT
**TGF-β**	ACTGGAGTTGTACGGCAGTG	GGATCCACTTCCAACCCAGG
**IL-1β**	TGCCACCTTTTGACAGTGATG	AAGGTCCACGGGAAAGACAC
**GAPDH**	TGAAGGTCGGTGTGAACGG	CGTGAGTGGAGTCATACTGGAA

## Data Availability

Data available on request due to restrictions. The data presented in this study are available on request from the corresponding author. The data are not publicly available due to privacy.

## Supplementary Materials

The following supporting information can be downloaded at: https://www.mdpi.com/article/10.3390/nu14030723/s1, Figure S1: Gating strategy and MLN results.
